# Protein and lipid expansion microscopy with trypsin and tyramide signal amplification for 3D imaging

**DOI:** 10.1038/s41598-023-48959-9

**Published:** 2023-12-08

**Authors:** Ueh-Ting Tim Wang, Xuejiao Tian, Yae-Huei Liou, Sue-Ping Lee, Hsiao-Tang Hu, Chieh-Han Lu, Po-Ting Lin, Ya-Jen Cheng, Peilin Chen, Bi-Chang Chen

**Affiliations:** 1 Affiliated Senior High School of National Taiwan Normal University, Taipei, 106348 Taiwan; 2 Research Center for Applied Sciences, Academia Sinica, Taipei, 11529 Taiwan; 3 Nano Science and Technology Program, Taiwan International Graduate Program, Academia Sinica and National Tsing Hua University, Taipei, 11529 Taiwan; 4 Department of Engineering and System Science, National Tsing Hua University, Hsinchu, 300 Taiwan; 5 Institute of Molecular Biology, Academia Sinica, Taipei, 11529 Taiwan; 6 Institute and Undergraduate Program of Electro-Optical Engineering, National Taiwan Normal University, Taipei, 116 Taiwan; 7 Neuroscience Program, NPAS, Academia Sinica, Taipei, 11529 Taiwan, ROC

**Keywords:** Microscopy, Fluorescence imaging

## Abstract

Expansion microscopy, whereby the relative positions of biomolecules are physically increased via hydrogel expansion, can be used to reveal ultrafine structures of cells under a conventional microscope. Despite its utility for achieving super-resolution imaging, expansion microscopy suffers a major drawback, namely reduced fluorescence signals caused by excessive proteolysis and swelling effects. This caveat results in a lower photon budget and disfavors fluorescence imaging over a large field of view that can cover an entire expanded cell, especially in 3D. In addition, the complex procedures and specialized reagents of expansion microscopy hinder its popularization. Here, we modify expansion microscopy by deploying trypsin digestion to reduce protein loss and tyramide signal amplification to enhance fluorescence signal for point-scanning-based imaging. We name our new methodology TT-ExM to indicate dual trypsin and tyramide treatments. TT-ExM may be applied for both antibody and lipid staining. TT-ExM displayed enhanced protein retention for endoplasmic reticulum and mitochondrial markers in COS-7 cell cultures. Importantly, TT-ExM-based lipid staining clearly revealed the complex 3D membrane structures in entire expanded cells. Through combined lipid and DNA staining, our TT-ExM methodology highlighted mitochondria by revealing their DNA and membrane structures in cytoplasm, as well as the lipid-rich structures formed via phase separation in nuclei at interphase. We also observed lipid-rich chromosome matrices in the mitotic cells. These high-quality 3D images demonstrate the practicality of TT-ExM. Thus, readily available reagents can be deployed in TT-ExM to significantly enhance fluorescence signals and generate high-quality and ultrafine-resolution images under confocal microscopy.

## Introduction

Fluorescence microscopy is a powerful approach for visualizing the fine cellular structures of organisms because of its high sensitivity and specificity. By means of antibody staining or fluorescent protein tagging, proteins in cells can be localized according to the distributions of emitted signals from fluorophores. However, the spatial resolution of fluorescence microscopy is limited to approximately half the wavelength of fluorescence signals, termed the Abbe limit, thereby preventing visualizations of ultrafine subcellular structures. With the invention of super-resolution techniques—such as stimulated emission depletion microscopy (STED)^1^, structured illumination microscopy (SIM)^2^, photoactivated localization microscopy (PALM)^3^, and stochastic optical reconstruction microscopy (STORM)^4^, it is now possible to image biological samples and circumvent the Abbe limit. Instead of improving optics to attain greater spatial resolution in super-resolution microscopy, expansion microscopy (ExM) was invented to bypass the optical diffraction limit by physically expanding biological samples in a hydrogel, enabling the effective resolution to be reduced to the nanometer scale under a conventional optical microscope^5–8^.

Originally, ExM was designed so that samples were expanded fourfold in one dimension^5^, which was called ×4 ExM. The protocol uses oligonucleotide-conjugated antibodies to recognize specific antigens and it reveals the location of the antibody-antigen complex based on fluorophore-conjugated complementary oligonucleotides^5^. The original protocol was then modified in various aspects. One was to increase the degree of expansion by modifying the hydrogel composition. Iterative ExM and ×10 ExM were subsequently developed by which samples were expanded tenfold or even higher in one dimension^9–11^. Very recently, the Magnify system was developed to crosslink different biomolecules to the hydrogel, allowing biological expansion of samples up to 11-fold^12^. Effective resolution was thereby improved from 250 to 63 nm (for ×4 expansion), to 25 nm (for ×10 expansion) or to ~ 22 nm (for ×11 expansion).

A second modification was to alter the fluorescence labeling approach. Given that using oligonucleotide-conjugated secondary antibodies for labeling is technically difficult and relatively expensive, alternative labeling strategies and traditional fluorescence-conjugated secondary antibodies have been adopted to replace them^6,13–16^. To prevent fluorophore loss, protocols for antibody staining after gelation and expansion have also been established^8,17–19^. However, it is still challenging to handle hydrogels for post-labeling and the caveat of fluorophore dilution still remains.

A third improvement to ExM is to use different proteases. Given that oligonucleotide-conjugated secondary antibodies are used to label the positions of antigens, the original protocol uses proteinase K to digest protein molecules as completely as possible, enabling free expansion of the fluorophore with the hydrogel^5^. However, since proteinase K is a very potent protease with broad substrate specificity, the peptide products it generates are excessively small and it impairs fluorophore retention when fluorophore-conjugated secondary antibodies are used. Thus, different proteases or a combination of heat and detergents have been investigated for ExM^13,16,20–22^. Although the expansion factors of other proteases are not better than those of proteinase K under the tested conditions, some proteases such as trypsin do display better fluorophore retention^21^.

The development and aforementioned modifications of ExM have already proven the value of ExM in imaging proteins, nucleic acids and lipids at the ultrafine structures of cells and tissues^5–7,9,23–26^. However, the reduction in fluorescence signals inherent to diluting fluorophore concentrations still poses an obvious problem. As the volume of a sample increases to at least 64- or 1000-fold (for ×4 and ×10 expansion, respectively), fluorophore concentrations concomitantly are diluted to 1/64 or 1/1000, which significantly reduces detectable fluorescence signals and impairs image quality, especially for 3D point-scan rendering. To enhance fluorophore signals and ensure their retention in expanded hydrogels, biotin-streptavidin complex^27^, trifunctional anchoring reagents^28^ and high-temperature homogenization^29^ have been incorporated into expansion microscopy methodologies to label protein molecules. However, the enhancement is still relatively limited. Thus, the dilution effect on fluorophore intensity remains one of the major drawbacks of expansion microscopy, hindering 3D microscopy at fine-scale sampling acquisition due to out-of-focus bleaching in the point-scanning process. Another challenge with this technique is associated with the complex nature of sample preparation and the specialized regents not commonly used in regular biological laboratories.

In this report, we aim to establish a simple protocol using readily available reagents, which are good characteristics for broad applications in biological labs. Even for ×4 expansion, the resolution can be increased from 250 to ~ 63 nm in a regular confocal laser scanner. If AiryScan2 is also used, such as LSM980 with AiryScan2, the resolution can be improved from 120 to ~ 40 nm, representing a significant improvement for general cell biology study. Based on all of these considerations and a literature review, here, we have established a modified ×4 ExM protocol using both trypsin digestion to enhance fluorophore retention and tyramide signal amplification to increase overall fluorescence signals. We call this new protocol expansion microscopy with trypsin digestion and tyramide signal amplification, i.e., TT-ExM in short.

Although trypsin has been previously successfully tested in ExM of *Caenorhabditis elegans*^21^, we noticed that the digestion buffer of trypsin in that previous study contained 0.5 M NaCl and 40 mM CaCl_2_, representing a high-salt condition. That condition is very different from the regular protocols, such as the passage of cultured cells using the trypsin–EDTA medium (Thermo Fisher Cat No. 25200) and sample preparation for mass spectrometry (Promega Cat No. V5111). Therefore, we modified the condition simply using culture-grade trypsin–EDTA in a low salt buffer to digest the stained cells embedded in a hydrogel. We found that compared to Proteinase K, the trypsin treatment indeed retained more fluorescence signals. Moreover, the subcellular structures of trypsin-digested cells—including nuclei, mitochondria, mitotic chromosomes and even chromatin in the nuclei of neurons—were isotropically extended with the entire cells.

For Tyramide Signal Amplification (TSA), fluorophore-conjugated tyramide is covalently linked to the side chain of tyrosine under peroxidase catalysis^30,31^. Therefore, it can increase the signal intensity of labeled fluorophores. The various combinations of peroxidase with the streptoavidin/biotin complex make it possible to label all kinds of biological molecules, including proteins, nucleic acids and lipids with fluorophores using TSA, which is the great advantage of using TSA. However, since TSA is an enzymatic reaction, one drawback of using TSA in expansion microscopy is that it is not suitable for single-molecule study. In the current report, we found that biotin-conjugated lipid molecules with TSA can clearly label all membranous structures and lipid-containing organelles of cells. Given that TSA labels fluorophores on proteins instead of lipids, the fluorophore-conjugated tyramides are well retained in the hydrogel after undergoing a regular ×4 expansion procedure. Thus, TSA exhibits particular advantages for labeling membrane or lipid-containing structures.

Here, we show that TT-ExM can be applied to both protein and lipid labeling, together with DNA counter-staining. The protocol is simple and easy to apply, yet it significantly increases fluorescence signals and retains fluorophores in the hydrogel-expanded samples. Entire 3D expanded cell images can be readily recorded using a confocal laser scanner, resulting in enhanced spatial resolution.

## Results and discussion

### The basic principle of TT-ExM

**Figure 1:**
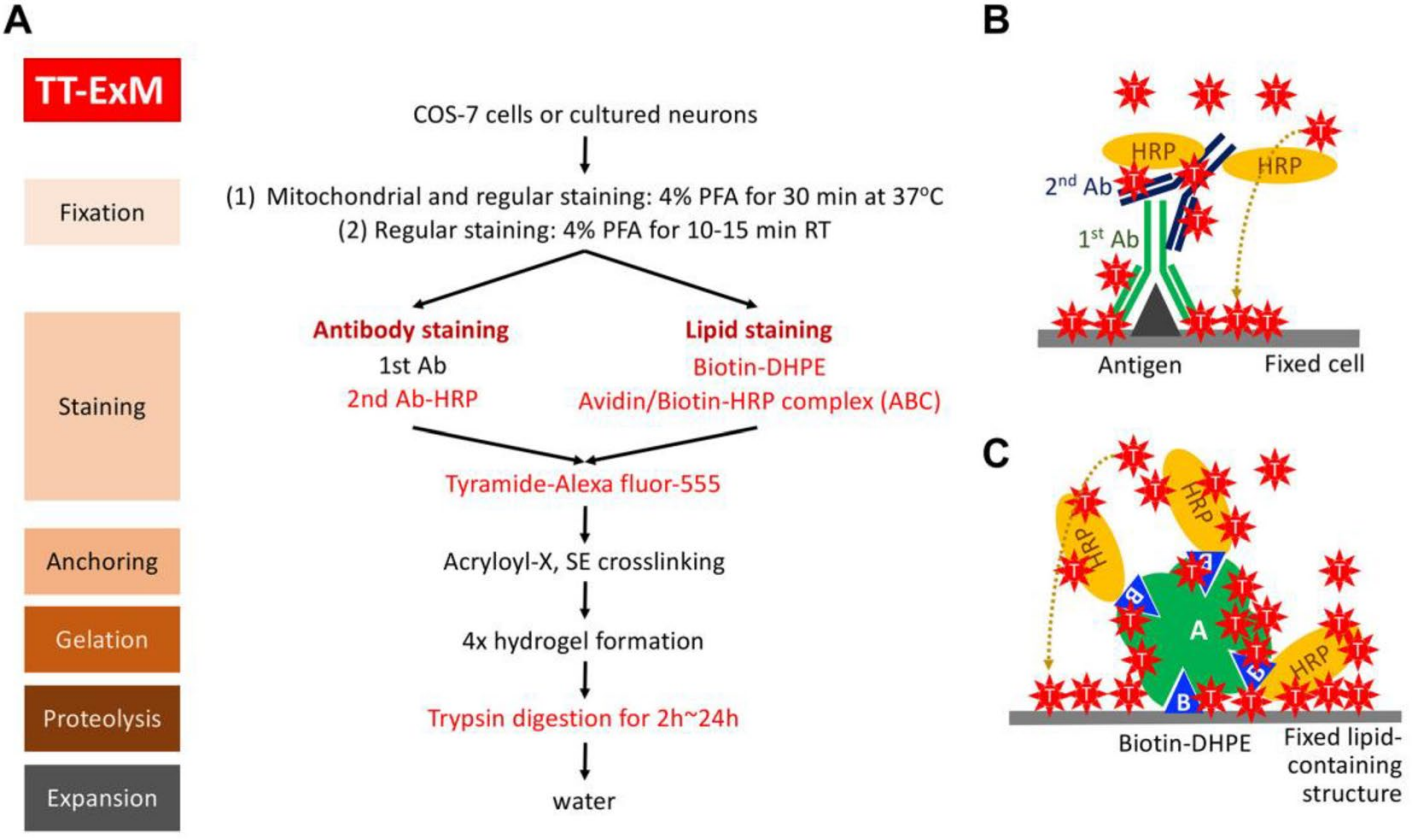
TT-ExM and tyramide signal amplification. (**A**) The major experimental steps of TT-ExM. The red font highlights the modified elements of a standard ExM protocol for TT-ExM. In brief, horseradish peroxidase (HRP) needs to be introduced into the staining step for subsequent tyramide signal amplification. Here, an HRP-conjugated secondary antibody and an avidin/biotin-HRP complex (ABC) kit are used for antibody and lipid staining, respectively. Trypsin replaces proteinase K for the proteolysis step. When the hydrogel is peeled from the coverslip, 2-h trypsin digestion is sufficient for a single-layer culture of COS-7 cells. For cultured neurons, to better preserve the structures of the long processes (i.e., axons and dendrites), we recommend immersing the entire gel on the coverslip in the trypsin solution and incubating it overnight or for up to 24 h until the gel freely separates from the coverslip. (**B**,**C**) The tyramide signal amplification (TSA) system used in the current study. (**B**) HRP-conjugated secondary antibody was used to label the primary antibody and to trigger the covalent linkage of Alexa fluor-555-conjugated tyramide to nearby protein molecules. (**C**) For biotin-DHPE-labeled cells, a premixed solution containing avidin and biotinylated HRP (i.e., ABC solution) was added to bind biotin-DHPE and induce conjugation of tyramide with the nearby proteins. Since each avidin molecule has four biotin-binding sites, by using ABC solution we could target more HRP to biotin-DHPE, thereby noticeably enhancing the fluorescent signals.

In order to enhance fluorescent signals in expansion microscopy, we made two modifications to the original protocol, as highlighted in red in the outline of TT-ExM depicted in Fig. 1A. First, we used the Tyramide Signal Amplification (TSA) system to overcome the dilution effect caused by hydrogel-mediated expansion, with horseradish peroxidase (HRP)-conjugated secondary antibody replacing the typical fluorophore-conjugated secondary antibody. In the presence of peroxidase and hydrogen peroxide, Alexa fluor-555-conjugated tyramide can be covalently linked to the side chain of tyrosine^32^, which enhances fluorescence signals (Fig. 1B). For biotinylated molecules, the avidin/biotin-HRP complex (ABC) can be applied to recognize and covalently conjugate tyramide to molecules or nearby proteins. For membrane and lipid staining, we used biotin-DHPE to label the membrane and lipid-containing structures in cells. Since ABC effectively recruits HRP molecules to biotinylated molecules, the signal enhancement attainable through ABC is much greater than for HRP-conjugated secondary antibodies (Fig. 1C). Thus, in general, the HRP-conjugated secondary antibody can be replaced with a combination of biotinylated secondary antibody and ABC^33^.

The second modification we did was to replace proteinase K with trypsin for proteolysis after the gelation step (Fig. 1A). Proteinase K is a broad-specificity protease routinely used in ExM protocols, which cuts at the carboxyl-terminal end of all aliphatic or aromatic amino acids (Table 1). Since the average frequency of all aliphatic or aromatic amino acids in protein molecules is 46.6% (Table 1), the average length of the proteolytic products using proteinase K is ~ 2.1 amino acid residues, which greatly reduces the possibility of anchoring fluorophore-conjugated peptides to the hydrogel and thus retain them there during expansion. This property of proteinase K as a potent protease therefore guarantees the proteolytic efficiency that allows anchored fluorophores to move freely with the hydrogel upon its subsequent expansion in water. In contrast to proteinase K, trypsin cuts specifically at the carboxyl-terminal end of lysine and arginine. The combined usage frequency of lysine and arginine in proteins is ~ 11.4% (Table 1). Therefore, the average length of the proteolytic products following trypsinization is ~ 8.8 amino acids. A previous study also demonstrated that trypsin treatment outperforms the standard proteinase K treatment under certain conditions in an immunohistochemistry experiment based on the fluorescence signal-to-noise ratio^21^. Accordingly, we anticipated that trypsinization would prove more efficient in terms of fluorophore retention in expansion microscopy.

**Table 1:** Comparison of proteinase K and trypsin digestion.

	Proteinase K	Trypsin (0.05%, Gibco 25,300–054), low-salt	Trypsin (0.05%, Gibco 25,300–054), high-salt
Working concentration of the protease	8 units/ml	0.015%	0.015%
Ingredients of the digestion buffer	Tris, pH 8.0, 50 mM EDTA, 1 mM Triton-X-100, 0.5% ^a^Guanidine HCl, 0.8 M	Tris, pH 8.0, 35 mM; EDTA, 0.7 mM; Triton-X-100, 0.35%	Tris, pH 8.0, 35 mM EDTA, 0.7 mM Triton-X-100, 0.35% NaCl, 0.5 M
Cleavage sites of the proteases	Aliphatic or aromatic amino acids, including leucine (7.6%), alanine (7.4%), valine (6.8%), isoleucine (3.8%), glycine (7.4%), phenylalanine (4%), proline (5%), tryptophan (1.3%) or tyrosine (3.3%)^b^	Lysine (7.2%) or arginine (4.2%)**	

^a^Guanidine HCl is a chaotropic agent, which can destroy intramolecular and intermolecular hydrogen bonds, thereby unraveling the three-dimensional structure of proteins and nucleic acids, which increases the hydrolytic efficiency of proteases. This chemical was only used in the proteinase K-based hydrolysis reaction, and not in the trypsin reaction.

^b^The percentages shown in parentheses after the amino acids represent the average frequency of use of each amino acid in vertebrates^44^, thereby reflecting the number of times that the protease cleaves a protein molecule with a length of 100 amino acids under ideal conditions.

### A combination of TSA and trypsinization retains convincing fluorescent signals in ExM

**Figure 2:**
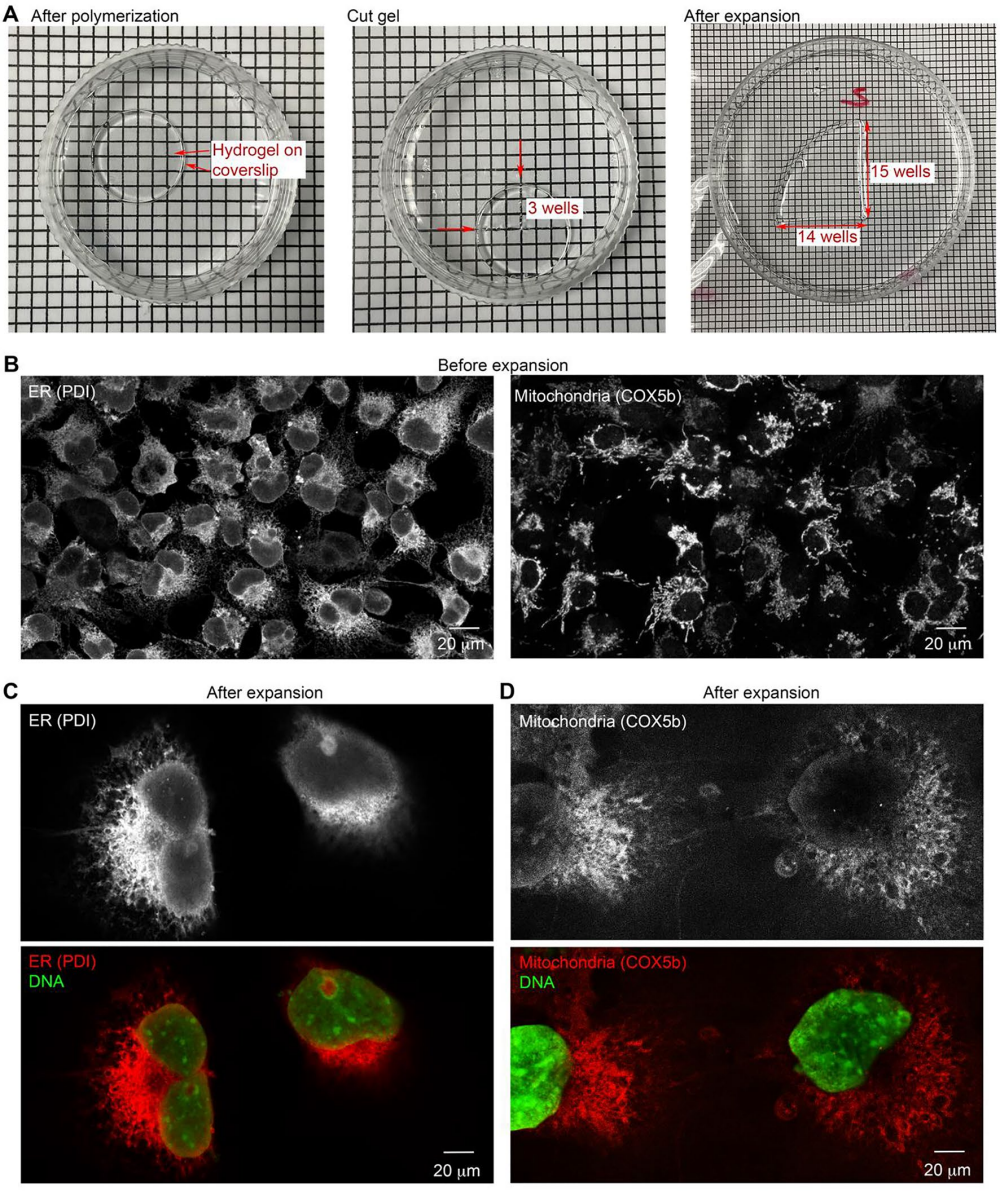
Deploying TT-ExM for high-quality immunostaining images. (**A**) More than fourfold expansion of our hydrogel. (**B**) Representative images before hydrogel expansion. COS-7 cells were immunostained using PDI and COX5B antibodies with TSA. (**C**,**D**) Representative images after hydrogel expansion. (**C**) PDI. (**D**) COX5B. The same ×20 objective lens and confocal microscope were used to acquire images from both before and after hydrogel expansion. DNA staining with PicoGreen was performed only after hydrogel expansion. Scale bars: 20 µm.

To evaluate the improvements achieved using TSA and trypsinization, first we tested antibodies that recognize protein disulfide isomerase (PDI, a marker of endoplasmic reticulum (ER)), and cytochrome c oxidase subunit 5B (COX5B, a mitochondrial marker), in TT-ExM. To prepare the hydrogel, we followed the protocol for 4× ExM^5^, but with some modifications^34^. The size of the gel was expanded ~ 4.8-fold (i.e., from 7.5 to 36.3 mm; Fig. 2A). Next, we examined immunoreactivities to confirm the successful application of the TSA system before hydrogel expansion (Fig. 2B). After trypsinization under a low-salt condition and expansion, a piece of hydrogel was removed and poststained with the DNA dye PicoGreen. For this experiment, a 20× objective lens was used to acquire fluorescent signals before and after expansion. From the resulting images, trypsinized COS-7 cells and their nuclei clearly extend evenly throughout the hydrogel (Fig. 2C,D). Note that the fields of view in Fig. 2B–D are the same, in which labeled cells were expanded ~ fourfold. Moreover, the scale bars shown in this and other figures of this manuscript have been drawn in a universal scale for ease of size comparison. Importantly, PDI immunoreactivity signals were well preserved and the images were easily acquired using an inverted LSM700 confocal laser-scanning microscope (Fig. 2C). Although COX5B signals were weaker than those of PDI upon expansion, we still acquired reasonably strong signals (Fig. 2D).

**Figure 3:**
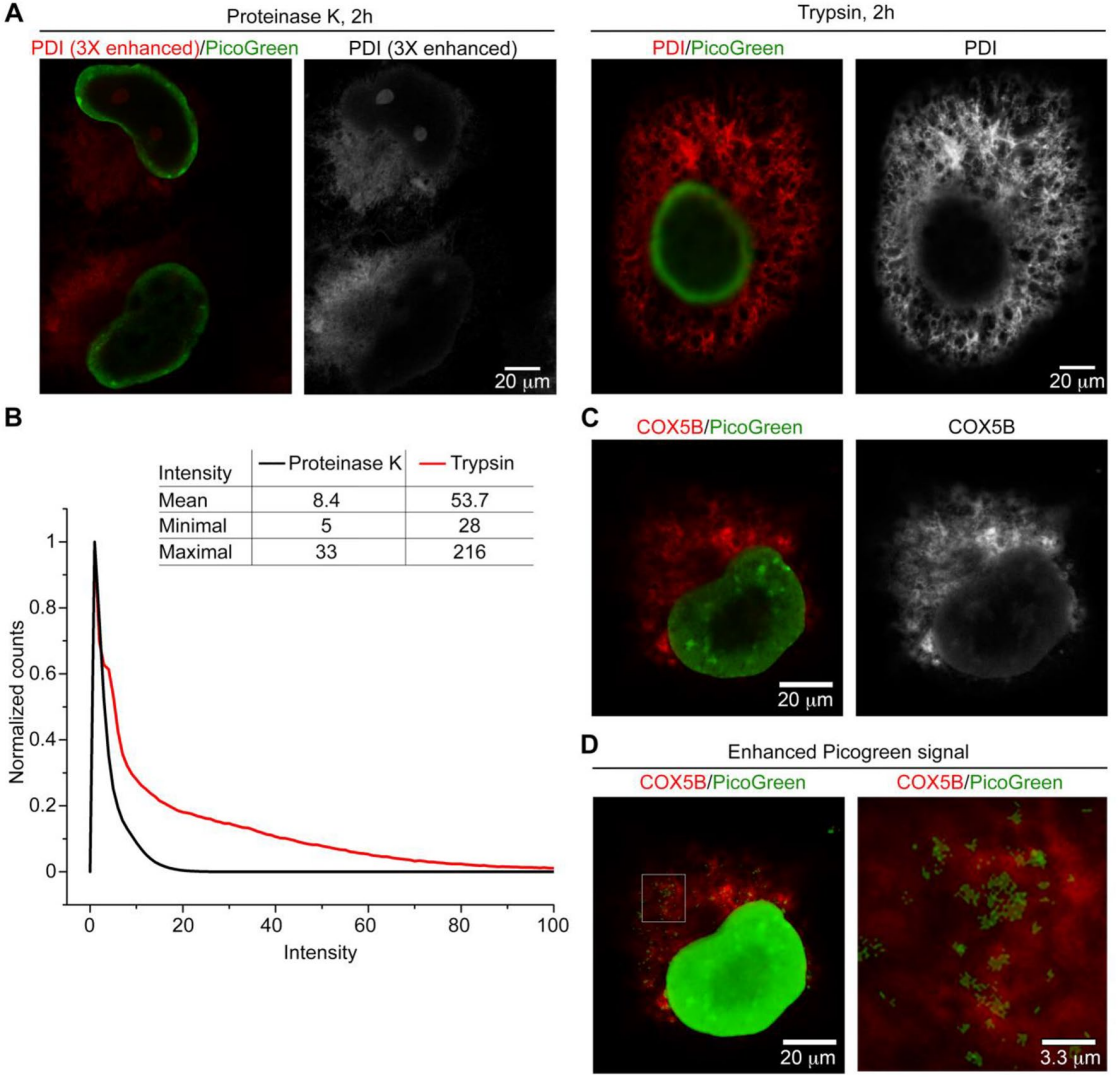
TT-ExM reveals fine-scale patterns of ER and mitochondria in COS-7 cells. (**A**) Comparison of 2-h proteinase K- or trypsin-based proteolysis. Two pieces of hydrogel were cut and peeled from the same hydrogel disc and subjected to digestion with either proteinase K or trypsin for 2 h. After poststaining using PicoGreen, the gels were observed using an LSM700 confocal system with a 20X objective lens. The images were acquired and processed under identical conditions to compare the effect of these two proteases. Since the PDI signals after proteinase K treatment were much weaker than those from trypsin digestion, the PDI signals following proteinase K digestion shown in the panel have been enhanced to enable visualization in the figure. (**B**) The PDI intensity of both images was calculated within the region thresholded by the “auto threshold” function in ImageJ. The intensity histograms have been normalized to 1 for comparison. (**C**) Visualization of mitochondrial signal in a COS-7 cell, as revealed by TT-ExM. (**D**) The same image as (**C**), except that PicoGreen signals have been greatly enhanced. The inset in the left panel has been enlarged at right, revealing that cytoplasmic PicoGreen puncta overlap with or are adjacent to COX5B immunoreactivity. Those PicoGreen puncta likely reflect mitochondrial DNA. Scale bars: (**A**), (**C**), (**D**) left, 20 µm; (**D**) right, 3.3 µm.

To evaluate our protocol further, we conducted a comparison of trypsin and proteinase K proteolysis treatments. We found that the immunoreactivity of proteinase K-treated cells was much weaker than for trypsinized COS-7 cells under the same experimental conditions (Fig. 3A). To present the images with similar contrast, we needed to vastly increase the brightness of the PDI fluorescence of proteinase K-treated cell images to compensate for the difference in signal intensity to those of trypsin-treated ones, indicating that fluorescence signals were indeed more strongly retained by trypsin treatment. The signal intensity distribution of the trypsinized cells displays a notable shift to the higher intensity region and a significantly increased mean intensity (Fig. 3B). We observed the mesh-like structure of ER (Fig. 3A, right) and some bagel-shaped mitochondria in our images achieved using only a 20× objective lens (Fig. 3C). When we enhanced the signals of PicoGreen-labeled nuclei to near saturation, we observed tiny puncta of PicoGreen signal in the cytoplasm. These signals primarily overlapped with or were adjacent to COX5B immunoreactivities (Fig. 3D), thus potentially representing mitochondrial DNA. Based on these results, we have demonstrated that our TT-ExM protocol noticeably enhances and retains fluorescence signals in hydrogels and that clear images can be easily acquired using a 20× objective lens under an inverted confocal microscope.

**Figure 4:**
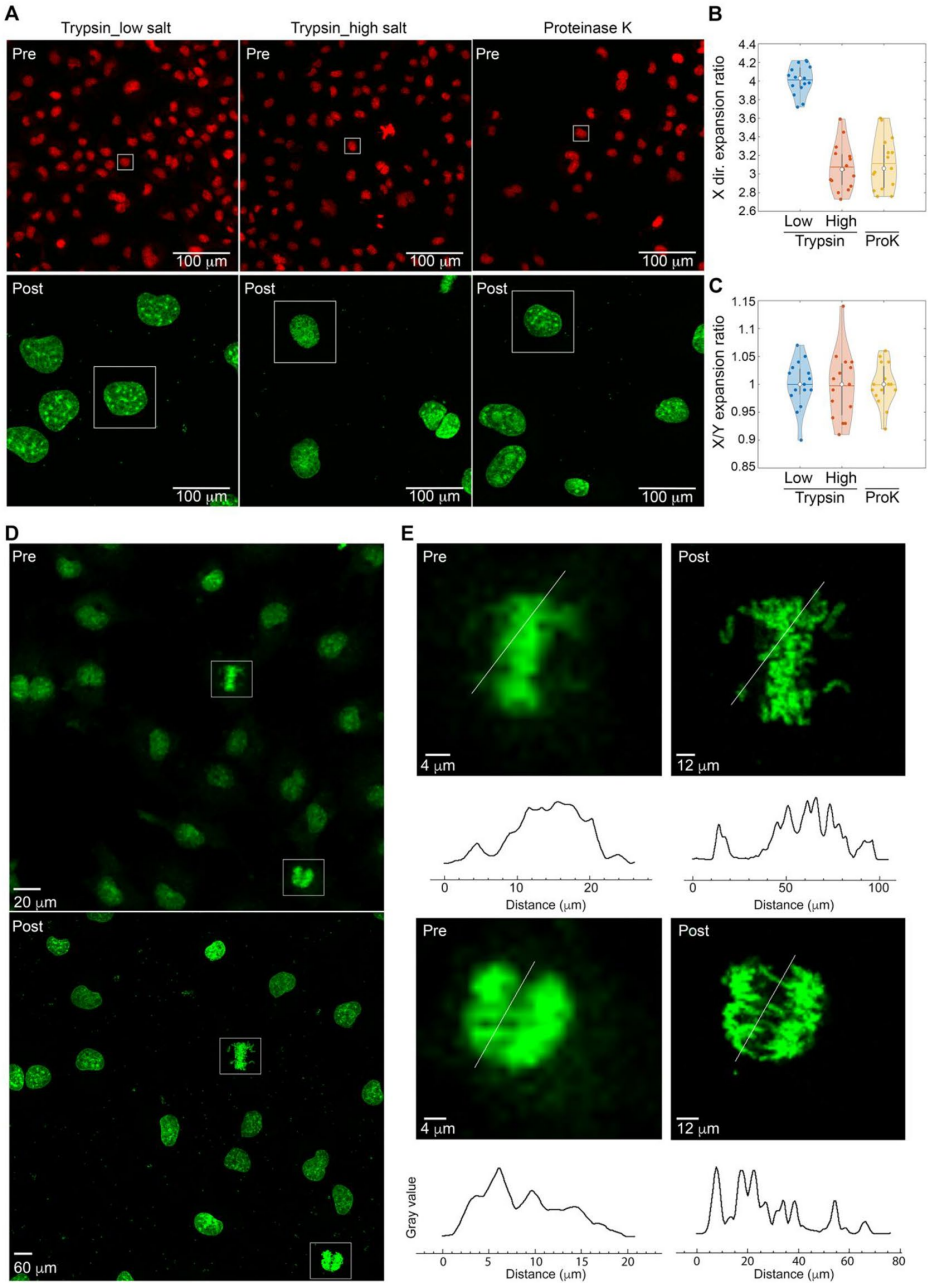
Isotropicity of TT-ExM assessed by quantitative analysis. (**A**) Images of the pre-expanded (upper row) and post-expanded (lower row) cells under different experimental conditions. The boxed cells are representative of those subjected to quantitative analysis. (**B**) The expansion ratio in the x-direction. The statistical distribution is plotted over 15 independent cells. (**C**) The ratio between the one-dimensional expansion ratio in the x and y directions reveals an isotropic expansion in the planar direction. (**D**) The same cells undergoing mitosis are identified in the pre-expansion image (upper) and post-expansion (below) images. (**E**) Comparison between the pre-expanded (left) and post-expanded (right) cells. Line profiles along the same cross section are presented below, showing a distinct resolution improvement after expansion under the same imaging condition. Scale bars: (**A**) 100 µm, (**D**) 20 µm for Pre- and 60 µm for Post- (E) 4 µm for Pre- and 12 µm for Post-.

To assess the effectiveness of TT-ExM, we quantitatively determined the expansion ratio between pre-and post-expanded cells. Here, we compared side-by-side the effects of trypsin in both low- and high-salt buffers and proteinase K. As shown in Fig. 4A, we selected images from the same cell under different experimental conditions for comparison. On the prerequisite that the samples are linearly expanded, the one-dimensional expansion ratio is plotted in Fig. 4B, showing that the low-salt trypsinated cells display significantly higher expansion ratio values than under the other two conditions. We then adopted a similar assessment to check the isotropicity of TT-ExM. In Fig. 4C, we show that the expansion ratio in the x and y direction does not differ significantly under different conditions. Thus, our analysis reveals that the low-salt environment employed in TT-ExM provides a higher expansion ratio than proteinase K while maintaining a homogeneous expansion. To further highlight the imaging resolution improvement acquired by a 10× NA0.45 objective lens through sample expansion, several mitotic cells were zoomed from Fig. 4D (pre- and post-expansion images) in Fig. 4E, where the distinct condensed chromosomes of post-expanded cells could be easily observed compared to those before expansion, demonstrating the power of breaking the diffraction limit confined by 10× NA 0.45 lens.

### Application of TT-ExM for lipid staining

**Figure 5:**
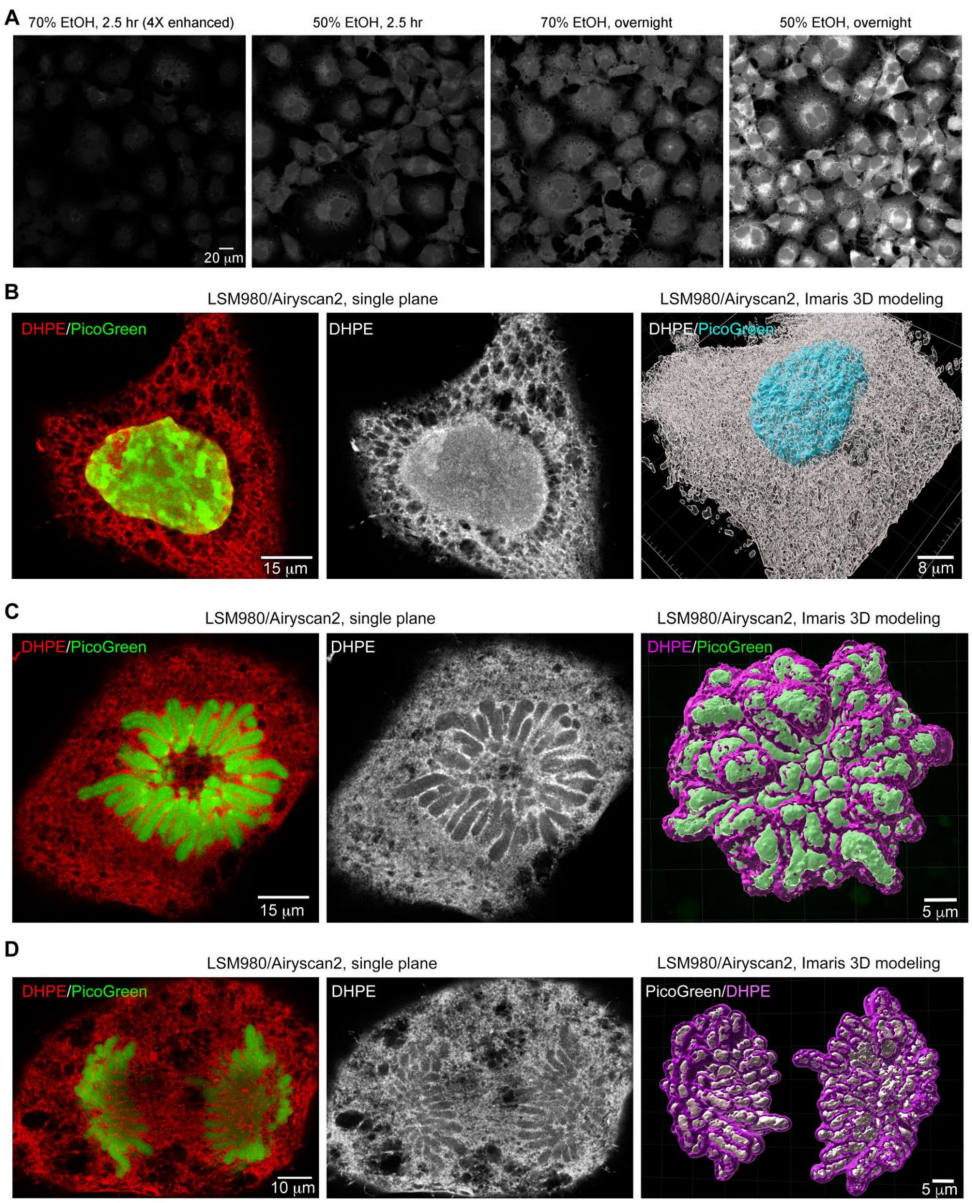
Biotin-DHPE staining highlights the complex lipid-containing structures in entire COS-7 cells. (**A**) Comparison of biotin-DHPE staining conditions. Two concentrations of ethanol, i.e., 50% and 70%, were used to dilute biotin-DHPE and cells were stained for either 150 min or overnight. Before expansion, the fluorescent signals from the four above-described conditions were recorded using the same confocal microscope, the same lens, and the same laser power. The results indicate that a combination of 50% ethanol and overnight incubation resulted in the best biotin-DHPE staining signals. Since the signals of the experimental group stained using 70% ethanol for 150 min were too weak for image visualization, the biotin-DHPE signals have been enhanced fourfold for the image in the panel. (**B**–**D**) Representative images of COS-7 cells acquired by super-high resolution LSM980 confocal microscopy with Airyscan2. The panels at left are single-plane color images of DHPE (red) and PicoGreen (green) signals. The middle panels show biotin-DHPE signals only. The panels at right are the results of Imaris 3D modeling. (**B**) Cell at interphase. (**C**) Cell at prometaphase. (**D**) Cell at anaphase. In the panel at right of (**B**), Imaris was used to model the surface of biotin-DHPE signals in an entire cell, excluding the nucleus. In (**C,D**), the panels at right highlight the interaction between chromosomes and lipid-containing materials. Scale bars: (**A**) 20 µm; (**B**) left, 15 µm; right, 8 µm; (**C**) left, 10 µm; right, 5 µm; (**D**) left, 15 µm; right, 5 µm.

Apart from applications in imaging protein distributions, it can also be important to visualize cellular structures by means of lipid staining. Biotin-DHPE can be used to label lipid-containing structures^35–37^, so we investigated if it can be applied to TT-ExM to stain the lipid structures of cells. We dissolved biotin-DHPE in chloroform and then diluted the mix with 70% or 50% ethanol for cell staining, with incubation times of 150 min or overnight. Among these four conditions, we found that the strongest biotin-DHPE fluorescent signals were obtained using 50% ethanol dilution and overnight incubation (Fig. 5A). Thus, we adopted this combination for subsequent experiments. Using a 20× objective lens, not only could we clearly visualize plasma membrane, but biotin-DHPE also labeled the intracellular membrane structures of COS-7 cells (Fig. 5A). To acquire images with a higher spatial resolution, we deployed an LSM980 confocal laser-scanning microscope with AiryScan2 and observed the nucleus and cytoplasmic mesh-like structures (presumably ER) in a cell at interphase using biotin-DHPE staining (Fig. 5B, left and middle). Intriguingly, we noticed that biotin-DHPE labeled the entire nucleus, including the nuclear matrix and even some nuclear aggregates lacking DNA (Fig. 5B, left and middle). We also performed sectioning in the z-plane and reconstructed a 3D image of an entire cell (except for the nucleus) using Imaris, which revealed the complex membrane organization of the entire cell (Fig. 5B, right). Our images depicted certain COS-7 cells undergoing cell division, with representative examples in prometaphase and anaphase shown in Fig. 5C and D, respectively. Significantly, the biotin-DHPE signal was enriched between the condensed chromosomes of the dividing cells (Fig. 5C,D), with these special biotin-DHPE-stained structures being attached to chromosomes (Fig. 5C,D, right panel), which is very similar to the pattern generated by staining for the cell proliferation marker Ki-67^38,39^. Therefore, we term this biotin-DHPE-stained structure as the “chromosome matrix”, which may be involved in chromosome organization and integrity during cell division.

Based on these images, we show that a combination of biotin-DHPE and TSA can be used in TT-ExM to label entire COS-7 cells, revealing many fine cellular details. Moreover, we have been able to observe the ultrafine structures of the cells under confocal microscopy.

### Observing mitochondria and nuclei in cultured neurons by means of TT-ExM

**Figure 6:**
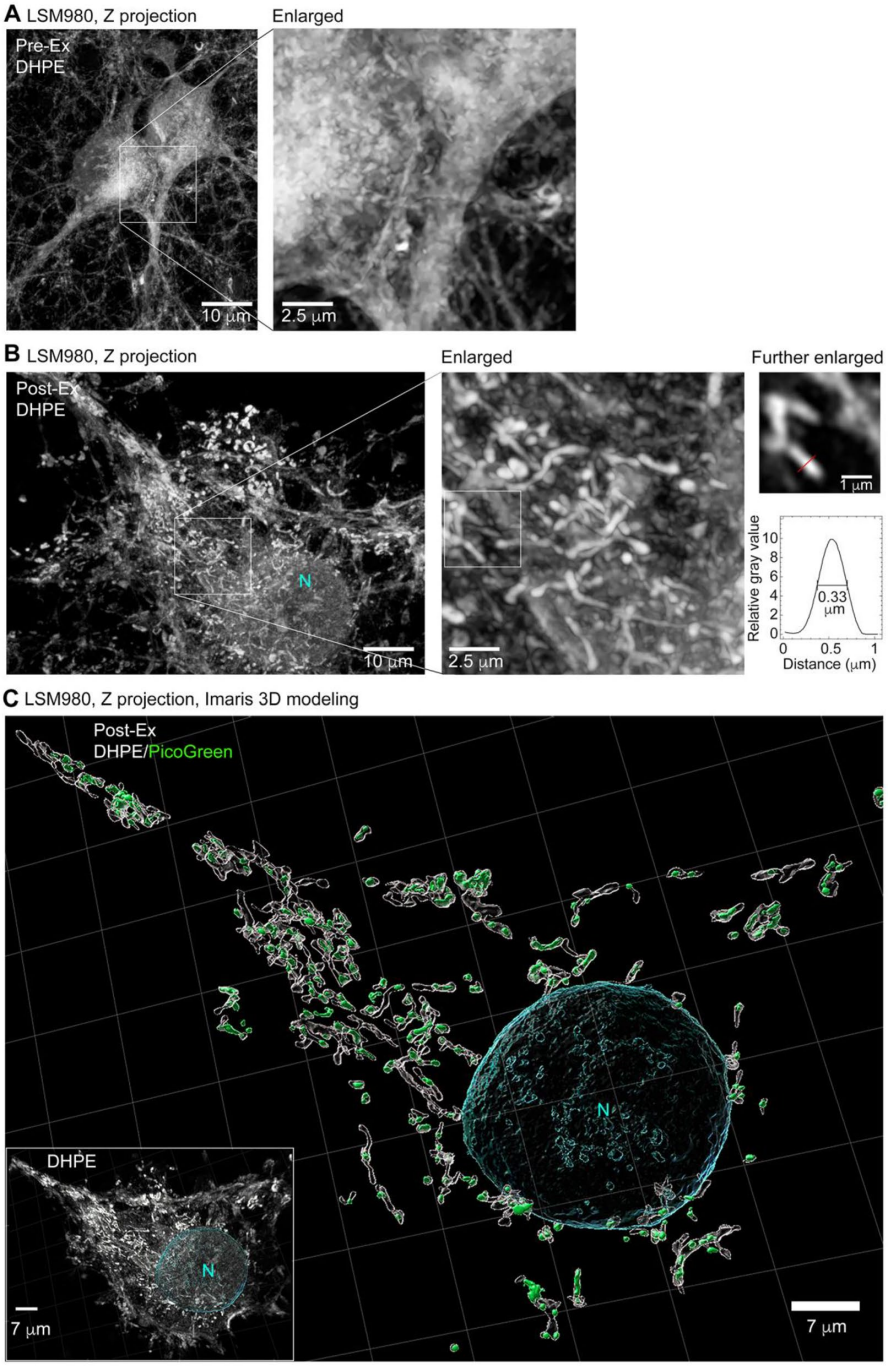
TT-ExM with biotin-DHPE and PicoGreen staining reveals clear mitochondrial morphology and patterning in cultured neurons. (**A**) Representative image of neurons before hydrogel expansion. An enlarged image shows the structures in two adjacent neurons. (**B**) The biotin-DHPE staining pattern after hydrogel expansion. The densely packed mitochondrial network can be observed in the enlarged image. The cross-section profile of one mitochondrion is shown in the inset, indicating a full width at half maximum of 0.33 µm. (**C**) The Z-series images from (**B**) were used for 3D modeling in Imaris. The position of the nucleus was determined by the PicoGreen signal, which was then masked to minimize its influence on viewing cytoplasmic DNA. The PicoGreen-positive puncta in the cytoplasm were always enwrapped by DHPE-positive tubular structures of ~ 0.5–1 µm in width. Based on these features, we speculate that these structures represent mitochondria. Scale bars: (**A**,**B**), 10 µm; (**C**) 7 µm.

In addition to studying COS-7 cells, we also endeavored to image primary cultured neurons prepared from mouse brains using TT-ExM and biotin-DHPE staining (Fig. 6). Neuronal cell bodies are approximately threefold smaller than COS-7 cells, so we only deployed LSM980 confocal microscopy with AiryScan2 to visualize neurons. We found that biotin-DHPE clearly stained the entire neurons, including their soma, dendrites and axons (Fig. 6A, before expansion). After hydrogel-based expansion, we clearly observed numerous DHPE-dense tubular structures in the cultured neurons (Fig. 6B, after expansion). Since these tiny tubular structures were ~ 0.5–1 µm in width and frequently harbored small PicoGreen-positive puncta (Fig. 6C), we speculate they represent mitochondria in the cultured neurons. Two enlarged views with the same field-of-view of pre- and post-expansion images shown in Fig. 6A and B demonstrate the gained spatial resolution after sample expansion under the same imaging conditions. A subplot in Fig. 6B shows a line-cut profile of the zoomed mitochondria having a diameter ~ 0.33 µm at full width at half maximum with an effective resolution of ~ 100 nm, matching with the dimension obtained from EM images for mitochondria inside the cultured mouse neurons, where ~ 100 nm width was observed^40^. The result shows that our TSA staining didn’t suffer from diffusion problem of amplification by the enzymatic reaction from the TSA process^41^, partly because we used lipid dye conjugated HRP system followed by expansion without much perturbation from the digestion treatment. Thus, TT-ExM deployed with lipid and DNA stainings can enable visualization of mitochondria in neurons.

**Figure 7:**
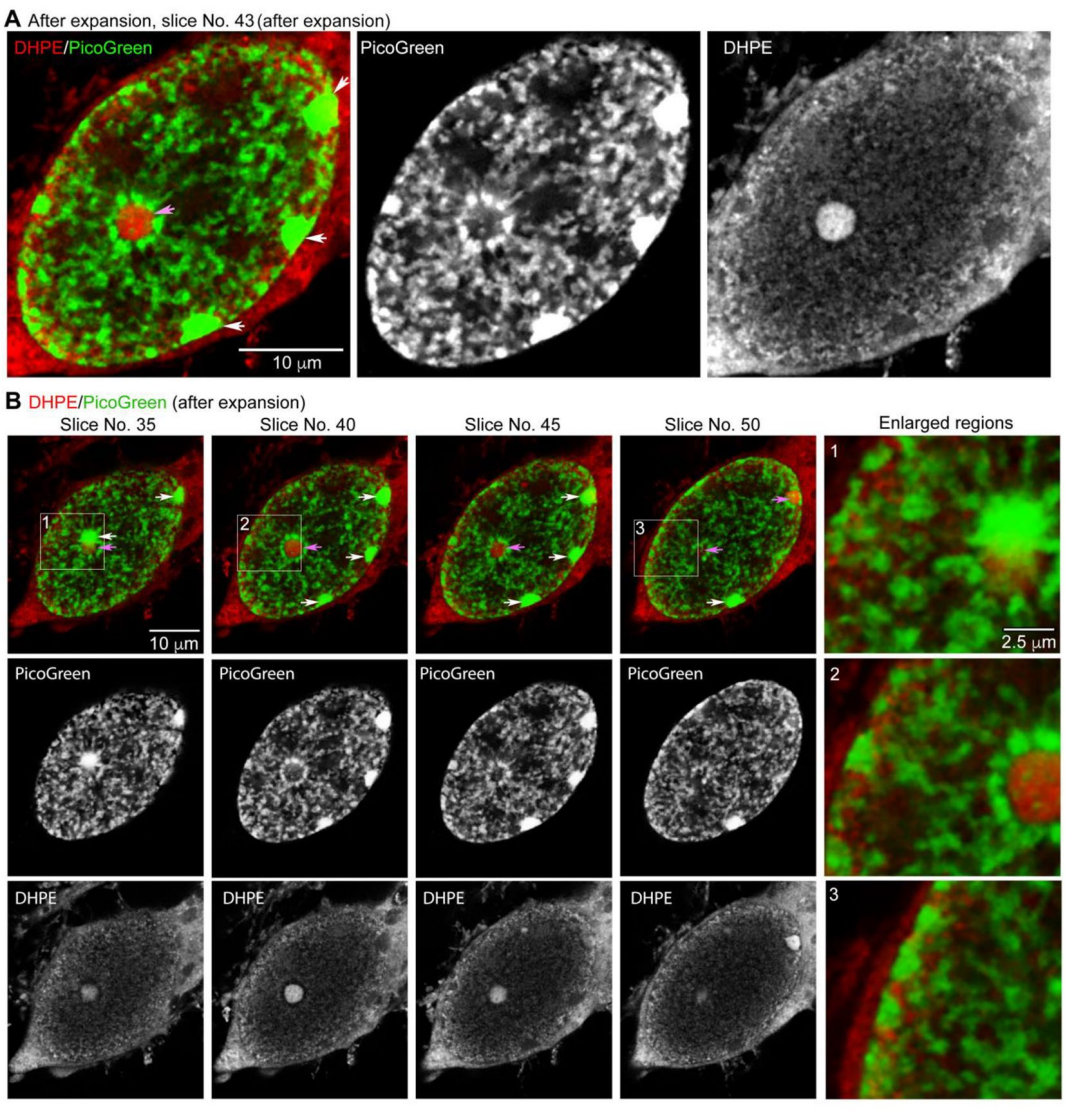
TT-ExM together with biotin-DHPE and PicoGreen staining reveals special nuclear structures and their organization in neurons. Z-series of images of a neuron were acquired using an LSM980 confocal microscope with AiryScan2 to monitor the staining patterns of biotin-DHPE and PicoGreen in the nucleus of a neuron after hydrogel expansion. (**A**) Enlarged images of the entire nucleus at slice No. 43 in the Z-series. (**B**) Images of slices #35, 40, 45 and 50 are shown. Three insets labeled #1, #2 and #3 in the panels have their enlarged images shown in the panel at right. Pink arrows point to the large biotin-DHPE-positive aggregates. White arrows indicate DNA aggregates. Scale bars: (**A**,**B**), 10 µm; (**B**) enlarged, 2.5 µm.

As for COS-7 cells, biotin-DHPE staining also highlighted the nuclear structure within neurons (Fig. 7A). Moreover, biotin-DHPE staining revealed numerous tiny droplets and some large condensates in the nucleus (Fig. 7A, B, pink arrows), which tended to be separate from but adjacent to chromosomal DNA (Fig. 7A, B). In particular, the large DHPE-positive condensates were always adjacent to large DNA aggregates, presumably representing the nucleolar structure (Fig. 7B, white arrows). In postmitotic neurons, DHPE-stained nuclear structures seemed to contribute to organizing chromosomal DNA and gene expression, unexpectedly revealing a special organization of lipid-containing structures in the nucleus.

## Conclusions

In summary, herein we present an improved protocol for expansion microscopy (TT-ExM) that enhances signal retention of protein and lipid cellular components and amplifies fluorescence signals. We adopted trypsin digestion for proteolysis of cultured cells, which engenders fewer cutting sites than the proteinase K treatment used in the original ExM method, resulting in more peptide fragments of organelles such as ER or mitochondria being anchored to the hydrogel matrix of TT-ExM. In addition, we deployed TSA coupled with HRP-conjugated secondary antibody to recruit more fluorophores, increasing fluorescence signal intensity. Since our aim is to increase fluorescence signals, we amplify the fluorescence signals by means of enzymatic reaction for demonstrating pre-expansion immunostaining TT-ExM. Doing so may result in the formation of a cloud of fluorophores around the target and engender labeling errors, but the diffusion of fluorophore signals could be reduced by modifying the reaction conditions^41^. Moreover, the labeling error could probably be minimized by post-expansion immunostaining since the sample expansion factor could compensate for it^42,43^. Due to the enzymatic reaction required for TSA, this probably hinders its applications in tissue expansion. Another caveat for TT-ExM is single-color enhancement by the TSA process. Hence, multiplex labeling and the combination of super-resolution techniques such as STORM or STED should be explored further for future applications^29^.

Moreover, by applying a combination of biotin-DHPE membrane dye, avidin/biotin-HRP complex and tyramide amplification, we were easily able to observe lipid-containing cellular components under a regular microscope, circumventing on oft-cited issue for previous expansion microscopy protocols due to their detergent-treatment steps. Through biotin-DHPE labeling, we uncovered in 3D a chromosome matrix at the chromosomal boundary during cell division. Therefore, TT-ExM also represents a convenient approach for investigating the nanoscale-level distributions of lipid-rich compartments in 3D during phase separation of biological systems by means of light microscopy.

Overall then, we have successfully applied TT-ExM to cultured cell systems for super-resolution imaging. By combining TT-ExM with DHPE staining, we have also achieved expansion microscopy for lipid visualization, which could potentially be useful for hydrogel-based expansion of large tissues, such as Drosophila melanogaster brain or zebrafish organs.

## Material and methods

### Reagents and material for cell culture

Dulbecco’s Modified Eagle Medium (DMEM), Gibco, 11965-084; Fetal bovine serum (FBS), HyClone, SH30084.03; l-Glutamine, Gibco, A29168-01; Penicillin/Streptomycin, Gibco, 15140-122; 10× PBS, pH 7.4, Lonza, 17-517Q, tenfold dilution before use; 0.05% Trypsin–EDTA, Gibco, 25300-054; poly-l-lysine, Sigma-Aldrich, P26360, 1 mg/ml; No. 1 coverslip, 15-mm in diameter, Fisher, 1942-10015.

### Cell preparation

The COS-7 cell line (ATCC, CRL-1651), an African green monkey kidney fibroblast-like cell line, was maintained in DMEM supplemented with 10% FBS, 1% penicillin/streptomycin and 2 mM glutamine in a 5% CO_2_ incubator at 37 °C. To prepare cell samples for expansion microscopy, COS-7 cells that grew to 100% confluency in a 10-cm culture dish were harvested and resuspended in 10 ml DMEM. The cell suspension (0.4 ml) was added into one well of a 12-well plate containing DMEM growth medium (1 ml/well) with a poly-l-lysine-coated coverslip. The cells were then placed back in a 5% CO_2_ incubator at 37 °C. One day later, cells were ready for immunostaining. 

### Reagents for cultured neurons

Dulbecco’s Modified Eagle Medium (DMEM), Gibco, 11960-044; Neurobasal medium, Gibco,12103-049; l-Glutamine, Gibco, A29168-01; l-Glutamic acid, Gibco, 11048; Penicillin/Streptomycin, Gibco, 15140-122; Hanks' Balanced Salt Solution (HBSS), Gibco, 14185-052; HEPES, Sigma, H4034; Papain, Sigma, P4762; EDTA, J.T. Baker, 8993-01; Calcium chloride (CaCl_2_), Merck, 1.02382; Deoxyribonuclease I (DNase I), Sigma, DN25; l-Cysteine, Sigma, C7352; Poly-l-lysine, Sigma, P2636; B-27 Supplement, Thermo Fisher, Fetal bovine serum (FBS), Gibco, 10437-028.

### Mediums/buffers for cultured neurons

HBSS buffer: 1× HBSS, Pen/Strep (1/100 dilution), 10 mM HEPES (PH = 7.3); poly-l-lysine buffer: 1 mg/ml poly-l-lysine; neuronal maintaining medium: DMEM/Neurobasal medium (1:1), B27 (1/50 dilution), Pen/Strep (1/100 dilution), l-Glutamine (1/100 dilution); neuronal plating medium: neuronal maintaining medium, 12.5 μM l-Glutamic acid; stop solution: DMEM, 10% FBS; Papain solution: 1× HBSS, 0.6 mg/ml papain, 0.5 mM EDTA, 1.5 mM CaCl_2_, 0.06% DNase I, 0.2 mg/ml cysteine.

### Ethics statement and ARRIVE guidelines

All animal experiments were performed with the approval of the Academia Sinica Institutional Animal Care and Utilization Committee (Protocol No. 18-10-1234) and in strict accordance with its guidelines and those of the Council of Agriculture Guidebook for the Care and Use of Laboratory Animals. All methods are reported following ARRIVE guidelines.

### Primary cultured cortical and hippocampal neuron

A pregnant C57BL/6 mouse was sacrificed by means of CO_2_ inhalation at embryonic day 18–19 and fetal pups of both sexes were killed by decapitation for neuronal cultures. Embryonic brains were dissected by sterilized forceps in laminar flow. Embryonic brains were put into cold HBSS and kept on ice. Cortical and hippocampal (Cx/Hp) regions were collected from embryonic brains and then washed with 10 ml cold HBSS 3 times. After HBSS wash, Cx/Hp were incubated with 2 ml papain solution at 37 °C for 20 min. The reaction was stopped by adding 10 ml stop solution. Cx/Hp neurons were dissociated by pipetting up and down several times gently and filtered by a 40 μm cell strainer. Neurons were collected by centrifuging at 900 rpm at RT for 5 min. The supernatant was discarded and neurons were suspended by adding 20 ml neuronal plating medium. Cell number was calculated by hemacytometer and 5 × 10^5^ cells were plated in a 12-well plate for each well (containing poly-l-lysine coated coverslip). Plating neurons were incubated in a 5% CO_2_ incubator at 37 °C. Five days later, half medium (0.5 ml) was replaced with 0.5 ml fresh neuronal maintaining medium for keeping cell growth. After incubation for five more days, neurons were subjected to lipid and DNA staining.

### Reagents for immunofluorescence staining

Paraformaldehyde (PFA), Merck, 1.04005.1000; Triton X-100, Sigma-Aldrich, T8787; Tris, VRW, 0826; Blocking reagent for immunostaining, Tyramide Signal Amplification kit, PerkinElmer, 581428; Cytochrome c oxidase subunit 5B (COX5B) rabbit polyclonal antibody, Proteintech, 11418-2-AP, 3–6 µg/ml; Protein disulfide isomerase (PDI) rabbit polyclonal antibody, Proteintech, 11245-1-AP, 1–2 µg/ml; Beta-tubulin mouse monoclonal antibody, Sigma-Aldrich, T4026, 6 µg/ml; Horseradish peroxidase (HRP)-conjugated anti-rabbit IgG, secondary antibody, Jackson ImmunoResearch Laboratory, 711-035-152, 1:500 dilution; Alexa fluor-488-conjugated anti-mouse IgG, secondary antibody, ThermoFisher, A28175, 1:500 dilution; Alexa fluor-555-conjugated anti-rabbit IgG, secondary antibody, ThermoFisher, A32732, 1:500 dilution; Alexa fluor-555-conjugated tyramide, Invitrogen, B40955, 1:100 dilution; Amplification diluent, PerkinElmer, FP1050; PicoGreen, ThermoFisher, P11495, 1:1000 dilution.

### Immunostaining with tyramide signal amplification

COS-7 cells that grew on coverslips were first washed with PBS three times and fixed with 4% PFA in PBS for 15 min at room temperature for regular staining. Alternatively, cells were washed three times with PBS pre-warmed to 37 °C and then fixed with 4% PFA in PBS pre-warmed to 37 °C for 30 min at 37 °C to observe mitochondria in cells. After washing twice with Tris-buffered saline (TBS), TBST (TBS + 0.3% Tritin-X-100) was added, before incubating for 10 min at room temperature for permeabilization. After rinsing once with TBST, 0.5% Blocking solution was added for 30 min at room temperature. Cells were then incubated with the primary antibodies diluted in blocking solution for 2 h at room temperature or overnight at 4 °C. After washing three times with TBST, secondary antibodies were added and incubated for 1.5 h at room temperature. After rinsing three times with TBST to remove unbound antibodies, Alexa fluor-555-conjugated tyramide was diluted 100-fold with amplification diluent and applied to cells for 10–20 min at room temperature. The cells were then rinsed three times with TBST to stop the reaction. Before crosslinking, immunofluorescence signals were checked under an inverted fluorescence microscope to confirm successful staining.

### Reagents for lipid staining

Paraformaldehyde (PFA), Merck, 1.04005.1000; 1× PBS; N-(Biotinoyl)-1,2-dihexadecanoyl-sn-glycero-3-phosphoethanolamine, triethylammonium salt (Biotin-DHPE), Biotium, 60022; VECTASTAIN ABC-HRP Kit, peroxidase (standard), Vector Laboratory, PK-4000; Alexa fluor-555-conjugated tyramide, Invitrogen, B40955, 1:100 dilution; Amplification diluent, PerkinElmer, FP1050; PicoGreen, ThermoFisher, P11495, 1:1000 dilution.

### Lipid staining using tyramide signal amplification

Biotin-DHPE was dissolved in chloroform at a concentration of 10 mg/ml. After washing three times with PBS, COS-7 cells or cultured neurons were fixed with 4% PFA in PBS for 15 min at room temperature. To observe mitochondria, the PBS and 4% PFA were pre-warmed to 37 °C directly before use. After washing three times with TBS, fixed cells were transferred to 70% or 50% ethanol containing 0.1 mg/ml biotin-DHPE and incubated for 2.5 h or overnight at room temperature. An equal amount of reagents A and B of an ABC-HRP Kit was mixed in TBS at a dilution of 1:50 for at least 30 min. Meanwhile, unbound biotin-DHPE was removed by extensively washing at room temperature. The premixed ABC solution was then added to biotin-DHPE-labeled cells for 1 h. After washing, Alexa fluor-555-conjugated tyramide was diluted 1:100 in amplification diluent and applied to cells for 15 min at room temperature. After extensive washing, the stained cells were then ready for anchoring to a hydrogel.

### Reagents for anchoring and making the acrylamide hydrogel

6-((acryloyl)amino)hexanoic acid (Acryloyl-X, SE), ThermoFisher, A-20770; Dimethyl sulfoxide (DMSO), Thermo Fisher, D12345; Sodium acrylate (SA), Sigma-Aldrich, 408220; Acrylamide (AA), Sigma-Aldrich, A9099; N,N′-methylenebisacrylamide (MBAA), ThermoFisher, 1551624; Ammonium persulfate (APS), Sigma-Aldrich, A9164; N,N,N,N,-Tetramethylethylenediamine (TEMED), Sigma-Aldrich, T7024; 4-Hydroxy-TEMPO, ALDRICH, 176141; Sodium Chloride, J.T.Baker, 3624-69.

### Anchoring

Before gelation, the stained cells were incubated with acryloyl-X, SE to crosslink protein molecules with the acrylamide gel. Acryloyl-X, SE was first dissolved in DMSO at a concentration of 10 mg/ml and then diluted to 0.1 mg/ml in PBS (pH = 7.4) right before adding it to the stained cells. The samples were incubated overnight at 4 °C for reaction. Directly before gelation, the samples were washed three times with PBS at room temperature to remove excess Acryloyl-X, SE.

### Gelation

To make sufficient acrylamide solution for six gel discs of 0.5 mm thickness and 15 mm diameter, 564 µl acrylamide monomer solution (containing 8.6% sodium acrylate, 2.5% acrylamide, 0.15% N,N′-methylenebisacrylamide and 11.7% sodium chloride in PBS) was mixed with 12 µl 10% TEMED, 6 µl 1% TEMPO, 6 µl water and 12 µl 10% APS. After extensively vortexing, 88 µl of the mixture was immediately added onto parafilm, which was placed in a 3.5-cm dish. After removing the extra PBS, a coverslip was placed on top of the acrylamide mixture with the side hosting cells facing down. Gelation was achieved at 37 °C for 2 h.

### Enzyme digestion and expansion of the hydrogel

Approximately a quarter of the hydrogel disc was cut with a scalpel and measured using graph paper. The gel was then gently picked up with a flat brush and placed in a solution containing a protease for hydrolysis. We tested two proteases, i.e., proteinase K and trypsin, incubated either for 2 h or overnight at room temperature. The concentrations of these two enzymes, the composition of the digestion buffer, and their cleavage sites are listed in Table 1. The digested hydrogel was completely immersed in double-distilled water in a 10-cm petridish. After at least 30 min, the double-distilled water was replaced with fresh water and this process was repeated at least three times. The size of the swollen hydrogel was again measured using graph paper.

### Poststaining with PicoGreen DNA dye

The expanded hydrogel was placed in water containing the DNA dye PicoGreen (1:1000) for at least 2 h at room temperature. To completely penetrate into nuclei, PicoGreen staining longer than overnight at 4 °C was required.

### Imaging using a LSM700 confocal laser scanner and image processing

Confocal laser scanner LSM700 was used to acquire images shown in Figs. 2B–D, 3A,C,D, 5A. An appropriate size (~ 0.5 × 1 cm) of the expanded hydrogel containing COS-7 cells was placed on a 24 × 50 mm coverslip with the side hosting cells facing down. The excess water was carefully removed. The expanded samples were then observed under an inverted confocal microscope LSM700 (Zeiss) with a Plan Apo 20×/NA = 0.8 DICII objective lens at room temperature. Non-expanded samples were directly mounted and observed using the same 20× objective lens under the same microscope for comparison with expanded samples. Fluorescence images were captured with Zen software 2009 (https://www.zeiss.com/microscopy/en/products/software.html) from the Zeiss Corporation. Single focal plane images were collected and then analyzed using ImageJ/Fiji software (V 2.1.0/1.53c, https://imagej.nih.gov/ij/index.html). The images were then converted into a format recognizable by general image software and assembled using Adobe Photoshop 2020.

### Imaging using a confocal laser scanner LSM980 system with Airyscan2 and image processing using Labkit and Imaris

For cultured neurons, the expanded gel was placed in a water-filled mold on a 0.17-mm-thick coverslip. Images shown in Figs. 5, 6 and 7 were recorded using a LSM980 laser scanning confocal microscope equipped with multiplex mode SR-4Y (2X sampling) and an Airyscan 2 detector at room temperature. A LD LCI Plan-Apochromate 40×/1.2 Imm Korr DIC M27 objective with working distance 0.41 mm was adopted for image acquisition. We applied 488 nm and 561 nm laser excitations for DNA and lipid signals, respectively. The 1844 × 1844 pixel images in the Z series were acquired at 0.2 µm intervals. Given that we scanned only the region of interest, the total scanning times of the images used in the current report were less than 8 min with Airyscan 2 multiplex mode SR-4Y mode. In order to minimize the sample drift issue, we have sandwiched the expanded gel between two coverslips with the varied thickness spacer according to the thickness of the expanded gel. Moreover, the coverslip has poly-l-lysine coated as a glue for the gel. The Z-series images were further processed to conduct 3D surface construction using Imaris 9.9.1 software with the machine learning Fiji-Labkit plugin for segmentation. The shortest distance and smooth options were chosen as Imaris parameter settings. The channel of interest was then directed to Labkit. In Labkit, the pencil tool was used to draw on images and identify "foreground" and "background" regions. This segmentation was sent back to Imaris to create the surface. In neurons, mitochondrion-like surfaces were defined by tubular lipid signals with a minimal object-object distance to DNA signals. The images were then converted into a format recognizable by general image software and assembled using Adobe Photoshop 2020.

To calculate the expansion ratios, LSM880 was used to acquire Fig. 4 for pre- and post- cell expansion by Plan-Apochromat 10×/0.45 objective with x, y, z (1.38 µm, 1.38 µm, 3 µm). We first identified and paired the same cell that appeared in the pre- and post-expansion images. The pre-expansion images were manually adjusted to match the post-expansion images, necessitating translation, rotation, and scaling. This series of operations generated a coarse expansion ratio between the pre-and post-images. To refine the expansion ratio, we used the "register image" function in Amira software from Thermo Scientific to examine the images further. Expansion ratios were calculated based on 15 cells. The average and median values are presented as horizontal bars and white circles, respectively, in Fig. 4B,C. The boundary of the distribution in the violin plots denotes the lower and upper bound of the dataset.

## Data Availability

The data that support the findings of this study are available from the corresponding author, B.C.C., upon reasonable request.
